# Lipophagy Dynamics in Hyperlipidemia Model ICR Mice Across Different High-Fat-Diet Feeding Durations

**DOI:** 10.3390/ijms27031573

**Published:** 2026-02-05

**Authors:** Shuang Xue, Xuan Guo, Qiao Wang, Xingtong Chen, Jinbiao Yang, Yunyue Zhou, Yukun Zhang, Wenying Niu

**Affiliations:** School of Basic Medical Sciences, Heilongjiang University of Chinese Medicine, Harbin 150040, China

**Keywords:** hyperlipidemia, lipophagy, autophagy, model validation, AMPK/mTOR

## Abstract

Hyperlipidemia (HLP) is a metabolic dysfunction marked by dysregulated lipid metabolism, which jeopardizes cardiovascular health. The function of autophagy modulated by the AMP-activated protein kinase (AMPK)/mammalian target of rapamycin (mTOR) pathway in HLP pathogenesis has not been fully elucidated. Thus, this study centered on the impacts of different feeding durations on HLP models. ICR mice were given a high-fat diet (HFD) to induce the model, with durations set at 3, 6, 9, 12, and 15 weeks. Body weight, liver and adipose organ indices, serum and hepatic lipid levels, and pathological changes (assessed by Oil Red O and HE staining) were measured. Related pathway markers were detected via immunofluorescence, quantitative real-time PCR (qPCR), and Western blotting. At week 9, the relative protein expression ratios of P-AMPK/AMPK, P-mTOR/mTOR, and P-ULK1/ULK1 were markedly reduced, while the expression levels of LC3Ⅱ/LC3Ⅰ and P62 proteins were notably elevated, exhibiting transient dysregulation characteristics and suggesting a potential optimal modeling time point. It clarifies the temporal pattern, core molecular mechanism, and critical turning point of abdominal adipose metabolic disorder induced by a high-fat diet (HFD) in ICR mice. This study offers a credible basis for the optimal duration of HLP modeling and in vivo animal experimental design.

## 1. Introduction

Hyperlipidemia is characterized as a disorder of lipid metabolism, with clinical manifestations of abnormally high serum total cholesterol (TC), triglycerides (TG), and low-density lipoprotein (LDL), or diminished high-density lipoprotein (HDL) concentrations [1]. Hyperlipidemia increases the incidence of stroke, diabetes mellitus, coronary heart disease, and a variety of other chronic diseases [2]. Alongside rapid economic growth, suboptimal eating patterns and inadequate physical exercise, the prevalence of hyperlipidemia has risen steadily annually. Among adult populations, hyperlipidemia serves as a critical risk factor determinant for cardiac and vascular conditions, especially atherosclerotic cardiovascular disorders [3]. Notably, LDL-C in peripheral blood circulation serves as a major contributor to atherosclerotic disorders. While lipid-lowering medications have enabled significant advances in the treatment of hyperlipidemia, the high prevalence of this disorder continues to place a substantial strain on global healthcare systems. Thus, achieving effective prevention and treatment of hyperlipidemia remains a formidable challenge to be addressed.

**Figure 1:**
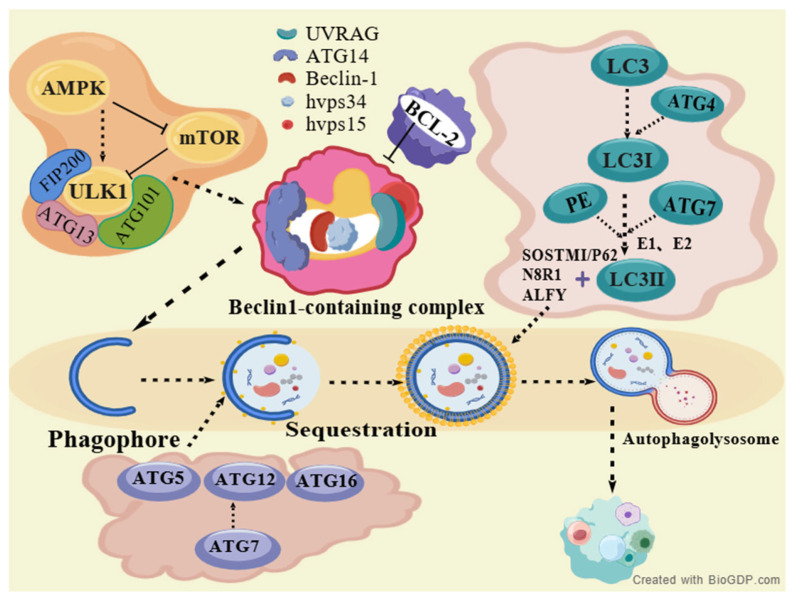
The procedure of autophagy regulated by the AMPK/mTOR signaling pathway created with BioGDP.com [12].

Autophagy is a highly conserved intracellular degradation and recycling process in eukaryotes, which has three lysosomal types (microautophagy, chaperone-mediated autophagy, macroautophagy; the latter is generally referred to as autophagy) [4]. AMPK/mTOR is a core autophagy regulator; mTOR (AMPK downstream), a negative regulator, suppresses autophagy upon activation [5]. Nutrient abundance sustains mTOR inhibition; starvation induces mTOR-ULK1 dissociation to activate autophagy [5]. ATG-modulated autophagy has four stages (Figure 1): (1) Initiation: AMPK inhibits mTORC1, relieving ULK1 complex (FIP200, ATG101, ULK1/2, ATG13) suppression to promote vesicle formation; ULK1 phosphorylates class ⅢPI3K complex I (Beclin-1, PIK3R4/p150, VPS34, ATG14/Barkor, AMBRA1) for phagophore nucleation [6,7]. (2) Elongation: ATG5-ATG12-ATG16 fuses with autophagosomal vesicles [8]; ATG4 cleaves LC3 to LC3-I, forming LC3-II (LC3-PE, membrane-bound) via ATG3/ATG7 + PE [9]. (3) Cargo sequestration: LC3-PE embeds in autophagosomal membranes, bridging selective autophagy via P62, NBR1, and NDP52 [10]. (4) Fusion: Lysosomes fuse with mature autophagosomes, secreting acid hydrolases for content degradation, nutrient/metabolite recycling, and autolysosome formation [11].

Lipophagy represents a form of macroautophagy, denoting the process by which autophagosomal vesicles specifically sequester lipid droplets—the principal intracellular organelles responsible for lipid storage. Lipophagy performs a critical role in regulating hepatic lipid metabolic activities, pancreas, and adipose tissue, thus liberating free fatty acids (FFAs) to act as a fuel source for mitochondrial β-oxidation [13]. With changes in human living environments, social environments, and lifestyles, investigations have indicated that lipophagy is not only intimately linked to liver diseases but also exerts an important function in metabolic perturbations, including hyperlipidemia, metabolic syndrome, atherosclerosis, and cancer. These findings have drawn researchers’ focus on the function of lipophagy in the pathogeny and management of illnesses. Therapy targeting lipophagy is poised to become a novel therapeutic approach while functioning as a key regulatory mediator for several related diseases. In particular, in adipose tissue, lipophagy maintains its normal physiological functions by regulating the dynamic balance of lipid droplets and acts as a key regulator of lipid metabolic homeostasis here. During the progression of hyperlipidemia, lipophagy dysfunction in adipose tissue exacerbates lipid accumulation and tissue inflammation, directly driving disease progression. Its functional status further serves as a key link between adipose tissue metabolic dysfunction and the development of hyperlipidemia.

Development of an optimized hyperlipidemia animal model enables more robust execution of animal experimental investigations into lipophagy-related mechanisms in hyperlipidemia, thus furnishing experimental evidence for the clinical prophylaxis and management of this disease. The selection of animal gender, modeling cycle, and modeling method varies when constructing a hyperlipidemia model: female mice are less likely to develop hyperlipidemia due to the effects of estrogen, whereas male mice have higher modeling success rates; in addition, the modeling duration of the high-fat-diet protocol varies, with the reported modeling periods in the existing literature covering 4, 6, 8, 12, and 14 weeks and other time frames, and the corresponding blood lipid levels exhibiting slight differences across different durations; available modeling methods include high-fat dietary administration, high-fat emulsion intragastric gavage, hydrocortisone intramuscular dosing, and poloxamer 407 intraperitoneal administration. Among these approaches, high-fat emulsion gavage is associated with a relatively high mortality rate; hydrocortisone intramuscular injection cannot fully simulate the actual pathological state of hyperlipidemia; and poloxamer 407 intraperitoneal injection is less commonly used due to its high cost. Thus, the high-fat-diet method is the most widely adopted one. It features simple operation and high modeling success rate, yet it requires long-term feeding with a relatively prolonged modeling duration [14,15,16,17,18]. In recent years, Chen et al. [19] devised an innovative model construction approach, wherein human apolipoprotein C3 (ApoC3)-transgenic C57BL/6 J mice were dosed with recombinant adeno-associated virus encoding PCSK9-D377Y (rAAV-PCSK9-D377Y) and simultaneously maintained on a high-cholesterol diet over 16 successive weeks, thus generating an innovative combined hyperlipidemia mouse model. Given the discrepancies in existing modeling cycles, and that most current studies on hyperlipidemia mouse models have focused predominantly on blood lipid profiles and hepatic histopathological characteristics, reports investigating the dynamic changes of lipophagy pathway-related proteins remain relatively scarce. Yet there remains a significant knowledge gap regarding the dynamic temporal progression of lipophagy dysfunction during high-fat-diet (HFD) intervention in vivo. The specific rules governing its progression from normal function to dysfunction, the key turning points, and the temporal correlation between this dysfunctional process and the pathological development of HFD-induced hyperlipidemia have not yet been systematically elucidated, which consequently renders the core mechanism underlying the role of lipophagy dysfunction in HFD-induced lipid metabolic disorders difficult to accurately decipher. Therefore, male ICR mice were employed in this study to systematically investigate the variations in various lipophagy detection indices across different modeling durations. The present study aimed to identify the optimal modeling duration for constructing a lipophagy-associated hyperlipidemia mouse model, thus furnishing a credible experimental model benchmark for subsequent explorations of the pathogenesis of hyperlipidemia and the formulation of therapeutic intervention methods.

## 2. Results

### 2.1. Overall Morphological Changes in Mice During High-Fat-Diet Feeding

Observation throughout each week of the experiment revealed that mice in the control group exhibited smooth and glossy fur, active movement, and normal fecal consistency. In contrast, model group mice exhibited fluffy, dull fur, significantly decreased activity, sluggish responsiveness, and mild loose stools.

### 2.2. Body Weight Changes in Mice During High-Fat-Diet Feeding

**Figure 2:**
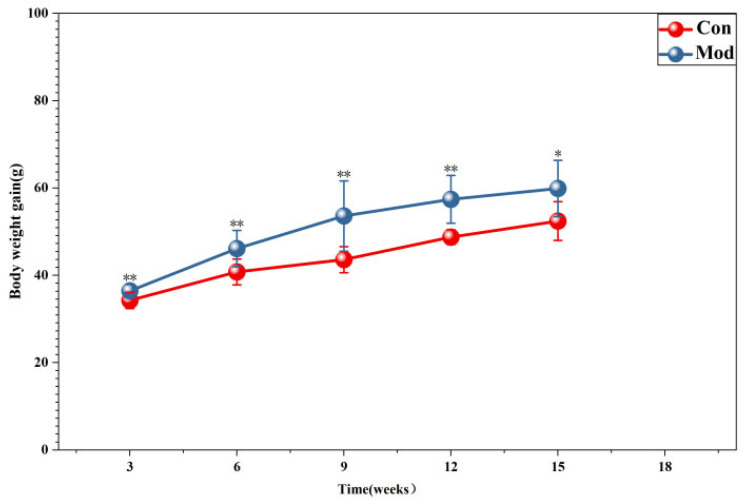
Body weight in mice. Data are presented as mean ± SD with eight mice per group. * *p* < 0.05, ** *p* < 0.01.

Body weight gain over the feeding period is presented in Figure 2 (data on initial body weight and serum lipid levels are provided in Supplementary Tables S2 and S3). Relative to the control group, high-fat-diet-fed mice exhibited significantly greater body weight gain (*p* < 0.01), with a statistically significant difference noted at week 15 (*p* < 0.05). Notably, the effect size for body weight between the model group and the blank control group was most pronounced at week 9 (Cohen’s d = 6.08; η^2^ = 0.43). Statistically significant differences in body weight were also observed at all other time points, as detailed in Table S1 (Supplementary Materials).

### 2.3. Comparison of Mouse Organ Indices During High-Fat-Diet Feeding

**Figure 3:**
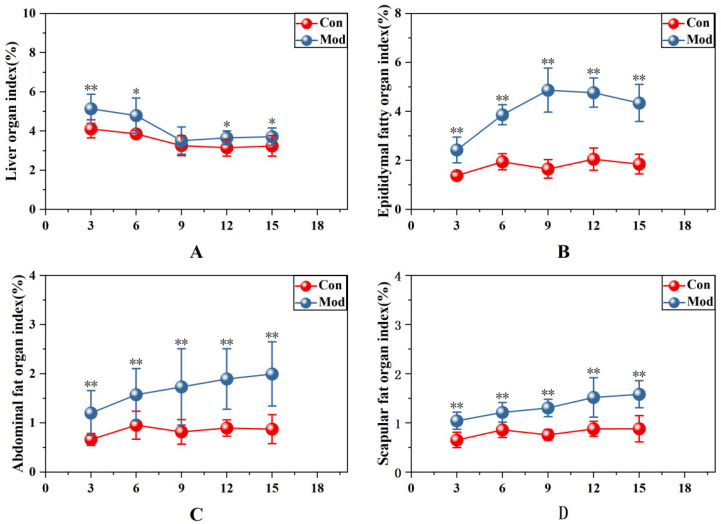
Organ indices of liver, epididymal fat, abdominal fat, and scapular fat in mice. (**A**) Hepatic organ coefficient; (**B**) epididymal adipose tissue index; (**C**) abdominal adipose coefficient; (**D**) scapular adipose tissue coefficient. Data are presented as mean ± SD with eight mice per group. * *p* < 0.05, ** *p* < 0.01.

Organ indices of the liver, epididymal, abdominal, and scapular adipose tissues among mice are presented in Figure 3. When compared with the control group, the hepatic index in the experimental model cohort was notably elevated at week 3 (*p* < 0.01) as well as weeks 6, 12, and 15 (*p* < 0.05), with no significant difference noted at week 9. The epididymal, abdominal, and scapular adipose tissue indices of the model group were all notably elevated (*p* < 0.01). The epididymal fat index peaked at week 9 and then gradually decreased. Notably, the effect sizes for epididymal, abdominal, and scapular adipose tissues reached the peak at week 9 (Cohen’s d = 0.670418, 0.57784, 0.145700; η^2^ = 0.88, 0.42, 0.77). The effect size differences of the above adipose tissues at all other time points are detailed in Table S1 (Supplementary Materials).

### 2.4. Assessment of Circulating Biochemical Markers in Experimental Mice During High-Fat-Diet Feeding

**Figure 4:**
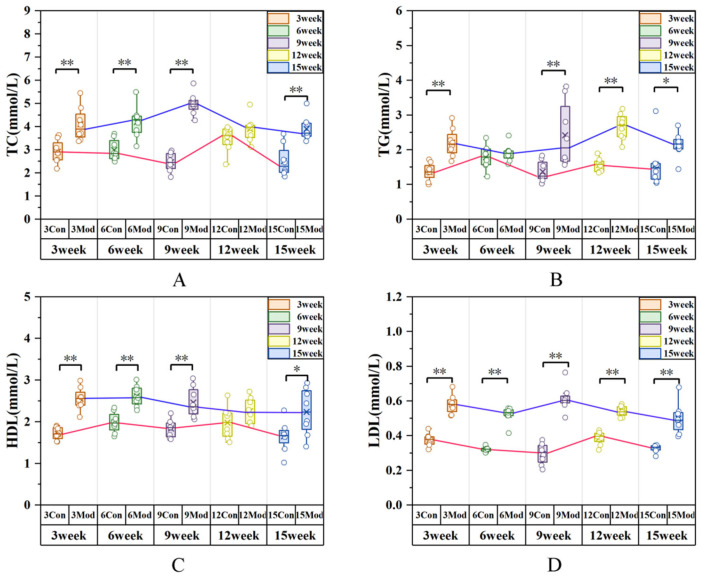
Circulating TC, TG, HDL, and LDL concentrations in mice across all groups. (**A**) CHO; (**B**) TG; (**C**) HDL; (**D**) LDL. Data are presented as mean ± SD with eight mice per group. * *p* < 0.05, ** *p* < 0.01.

Serum biochemical indices of mice are presented in Figure 4. Compared with the control group, the levels of total cholesterol (TC) and high-density lipoprotein (HDL) in the model group were significantly elevated at 3, 6, 9 and 15 weeks (*p* < 0.01), with a slight increase at 12 weeks that was not statistically significant. The triglyceride (TG) levels in the model group were significantly elevated at 3, 9, 12, and 15 weeks (*p* < 0.01, *p* < 0.05), while an increase was observed at 6 weeks without statistical significance. The low-density lipoprotein (LDL) levels were significantly elevated in all model groups at all time points (*p* < 0.01). At week 9, the effect sizes of serum TC, TG, HDL, and LDL between the blank group and the model group were (Cohen’s d = 0.435262, 0.688308, 0.294977, 0.066764; η^2^ = 0.91, 0.39, 0.56, 0.86). The effect sizes of the remaining groups are presented in Table S1 (Supplementary Materials).

### 2.5. Comparison of Total Cholesterol (TC) and Triglyceride (TG) Contents in Mice During High-Fat-Diet Feeding

**Figure 5:**
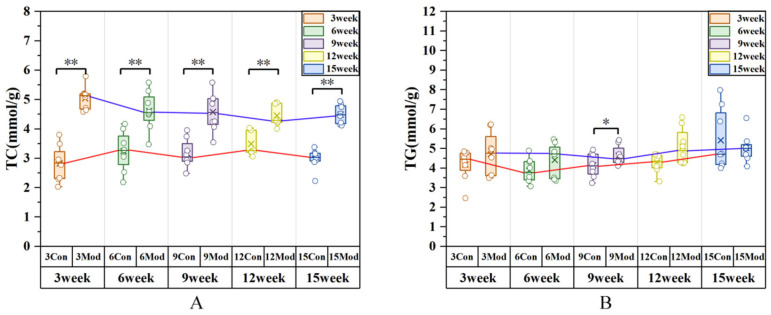
TC and triglyceride TG levels in mice across all groups. (**A**) Liver TC content; (**B**) liver TG content. Data are presented as mean ± SD with eight mice per group. * *p* < 0.05, ** *p* < 0.01.

Liver-derived TC and TG abundances in experimental mice are presented in Figure 5. Against the Baseline Arm, the model group’s liver TC content was significantly higher at all time points (*p* < 0.01; Cohen’s d = 0.58; η^2^ = 0.63). The model group’s liver TG content was elevated above the values of the reference cohort, accompanied by a statistically significant difference noted at week 9 (*p* < 0.05; Cohen’s d = 0.55; η^2^ = 0.18); the levels in the remaining groups were slightly increased with no statistical significance.

### 2.6. Comparison of Oil Red O Staining of the Liver and H&E Staining of Adipose Tissues in Mice During High-Fat-Diet Feeding

**Figure 6:**
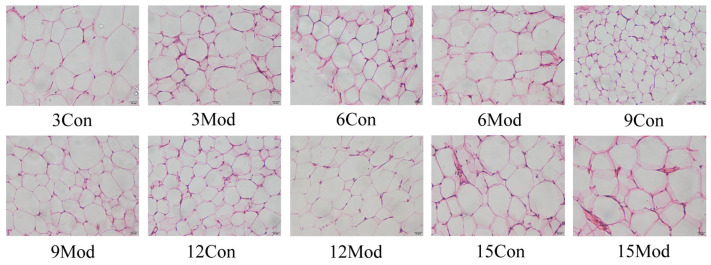
HE murine tissue staining abdominal adipose tissue (400×).

Figure 6 presents the HE staining results of iWAT from each group. Throughout the experimental period, adipocytes exhibited distinct stage-specific changes in morphology and volume: in the control group, adipocytes maintained the typical morphology of mature adipocytes at weeks 3, 6, 9, 12, and 15, with large vacuolar cytoplasm, nuclei compressed to the cell periphery, uniform size, and orderly arrangement, and notably, adipocytes in the 9-week control group were significantly smaller than those at other time points; in contrast, the model group showed progressive pathological evolution: at week 3, adipocyte volume was slightly larger than that of the contemporaneous control group but morphology remained relatively regular; at week 6, adipocyte volume increased significantly with prominent cytoplasmic vacuolization, indicating excessive lipid accumulation; at weeks 9 and 12, adipocyte volume was relatively reduced, accompanied by irregular cell morphology, nuclear displacement, and increased vacuolization; and by week 15, adipocyte volume increased again with disordered arrangement, suggesting a sustained progression of pathological processes.

**Figure 7:**
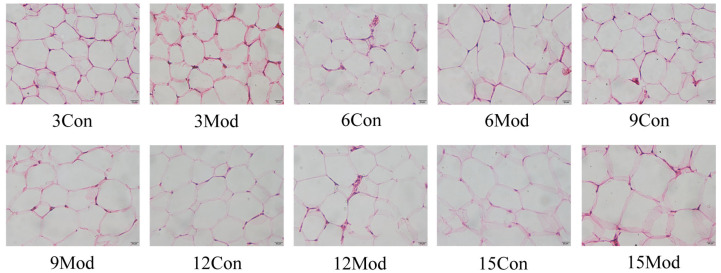
H&E staining of mouse epididymal adipose tissue (400×).

Figure 7 shows the HE staining findings of epididymal white adipose tissue (eWAT) from each group. Throughout the experimental period, adipocytes in all control groups maintained a regular round or polygonal morphology with uniform size, and their cytoplasm was filled with large unilocular lipid droplets that compressed the nuclei to the cell periphery, forming a characteristic flattened nuclear shape, with no obvious pathological changes observed. In contrast, the eWAT of the model group exhibited progressive pathological abnormalities: at week 3, adipocytes were already slightly hypertrophied compared with the contemporaneous control group, with subtle size heterogeneity; by week 6, hypertrophy became more pronounced, with irregular cell morphology, marked size variation, and continuous expansion of lipid droplets; at weeks 9 and 12, the hypertrophic state persisted, and the degree of nuclear flattening further increased, reflecting ongoing lipid accumulation; by week 15, adipocyte volume reached its maximum, with severely disordered cellular arrangement and extensive cytoplasmic vacuolization, suggesting a sustained progression of pathological processes underlying adipose tissue dysfunction.

**Figure 8:**
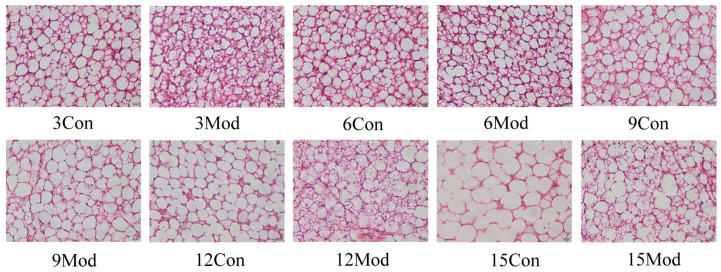
Mouse brown adipose tissue HE staining (400×).

Figure 8 presents the HE staining results of brown adipose tissue (BAT) from each group. In the control group, BAT maintained its typical normal structure at weeks 3 and 6, with adipocytes showing a regular polygonal morphology, cytoplasm filled with densely distributed small multilocular lipid droplets, and cells arranged compactly and orderly. By weeks 9 and 12, however, the control group exhibited clear signs of phenotypic transition: lipid droplets fused extensively into large vacuolar structures, the characteristic multilocular phenotype of BAT was largely lost, cells became extremely hypertrophic, and their arrangement became loose and disordered, indicating an obvious browning-to-whitening transformation; this whitening phenotype persisted at week 15, with adipocytes maintaining large vacuolar cytoplasm and loose cellular organization. In the model group, brown adipocyte volume increased progressively with prolonged feeding, accompanied by increasingly discellular arrangement. Notably, by week 9, massive fusion of lipid droplets into large vacuoles had already occurred, the typical multilocular phenotype was almost completely lost, and cells became extremely enlarged with loose, disorganized arrangement, demonstrating a prominent browning-to-whitening transformation that was more severe and appeared earlier than in the control group. By week 15, the whitening phenotype in the model group was further exacerbated, with adipocytes showing larger vacuolar structures and more severe disorganization compared to the control group at the same time point.

**Figure 9:**
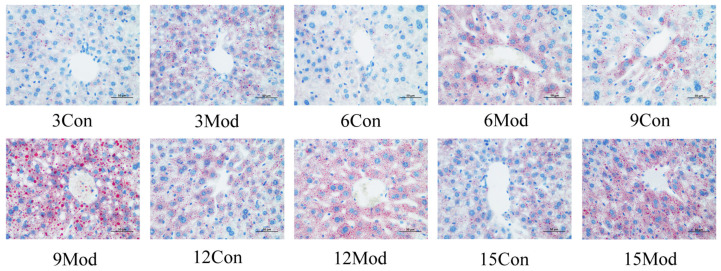
Oil Red O hepatic tissue stain (400×).

Figure 9 shows Oil Red O staining results of mouse hepatic tissues from all groups. In the control group, liver tissues exhibited minimal to no red lipid droplet deposition at weeks 3 and 6, with hepatocytes maintaining regular polygonal morphology and clear cytoplasmic boundaries. From week 9 onward, subtle lipid droplet deposition began to increase gradually, yet hepatocytes retained their orderly arrangement and intact structure, with no overt steatosis observed through week 15. In stark contrast, the model group displayed a progressive aggravation of hepatic lipid deposition over the experimental period: at week 3, sparse lipid droplets were already detectable; by week 6, deposition increased significantly, with small, scattered lipid foci appearing; at week 9, lipid droplet deposition reached its most severe level, with extensive, dense red lipid droplets filling the cytoplasm and forming large focal accumulations, accompanied by obvious hepatocyte swelling and distorted cellular arrangement; by week 12, lipid deposition showed a slight reduction, and at week 15, it remained less severe than at week 9, which may reflect a compensatory metabolic response in the liver.

### 2.7. Comparison of Immunofluorescence of P-AMPK, P-ULK1, and Beclin-1 Proteins in Mouse Abdominal Adipose Tissue During High-Fat-Diet Feeding

**Figure 10:**
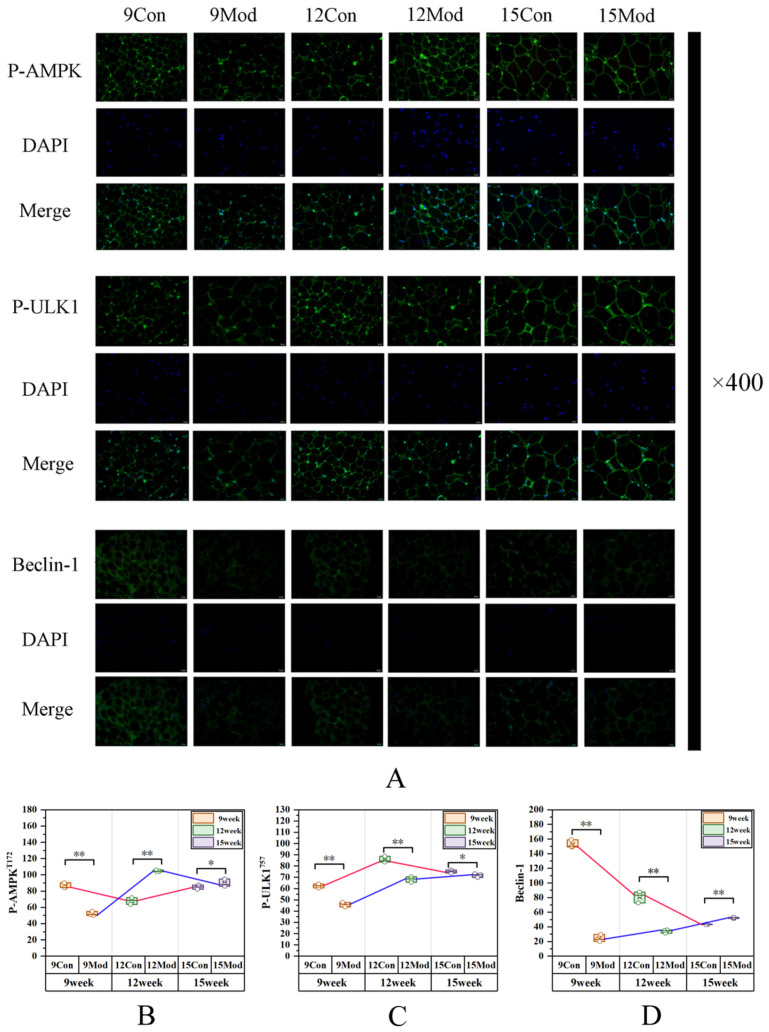
Immunofluorescence analysis of P-AMPK, P-ULK1, and Beclin-1 in mouse abdominal adipose tissue. (**A**) Immunofluorescence images of P-AMPK, P-ULK1, and Beclin-1; (**B**) quantitative analysis results of P-AMPK fluorescence intensity, Scar bar: 20 μm; (**C**) quantitative analysis results of P-ULK1 fluorescence intensity; (**D**) quantitative analysis results of Beclin-1 fluorescence intensity. Data are presented as mean ± SD with three mice per group. * *p* < 0.05, ** *p* < 0.01.

Figure 10 shows immunofluorescence expression of p-AMPK, p-ULK1, and Beclin-1 in mouse abdominal adipose tissue. Relative to the control group, model group p-AMPK fluorescence intensity was significantly reduced at week 9 (*p* < 0.01; Cohen’s d = 3.04; η^2^ = 0.98), with significant elevation at weeks 12 (*p* < 0.01) and 15 (*p* < 0.05). The model group’s *p*-ULK1 fluorescence intensity was overall notably reduced (*p* < 0.01), with a further marked reduction noted at week 15 (*p* < 0.05). Beclin-1 fluorescence intensity in the model group was markedly decreased at time points 9 and 12 (*p* < 0.01) but significantly elevated at week 15 (*p* < 0.01). At week 9, the effect sizes of P-ULK1 and Beclin-1 protein expression between the blank group and the model group were (Cohen’s d = 1.88, 5.17; η^2^ = 0.97, 1.00). The effect sizes of the other groups are presented in Table S1 (Supplementary Materials).

### 2.8. Western Blot Assay of AMPK/mTOR Signaling Pathway Proteins in Mouse Abdominal Adipose Tissue During High-Fat-Diet Feeding

**Figure 11:**
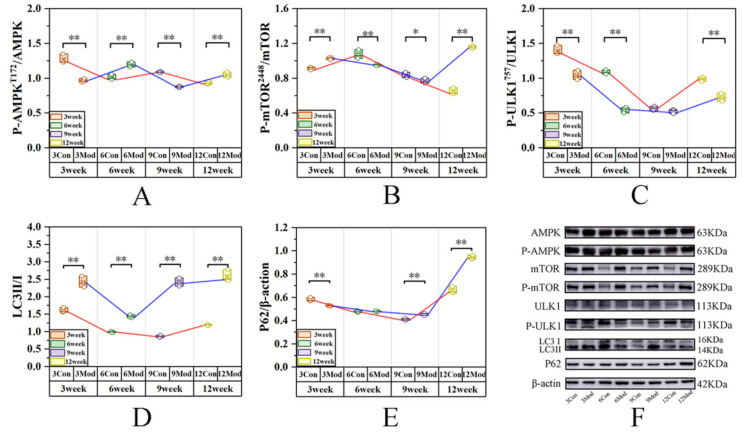
Western blot assay of P-AMPK, AMPK, P-mTOR, mTOR, P-ULK1, ULK1, LC3II, LC3I, and P62 expression in mouse abdominal adipose tissue. (**A**) Quantitative gray value analysis for the P-AMPK/AMPK ratio; (**B**) for the P-mTOR/mTOR ratio; (**C**) for the P-ULK1/ULK1 ratio; (**D**) for the LC3II/LC3I ratio; (**E**) quantitative gray value analysis of P62 expression; (**F**) representative Western blot bands of the aforementioned proteins. Data are presented as mean ± SD with three mice per group. * *p* < 0.05, ** *p* < 0.01.

Figure 11 depicts the expression of p-AMPK, AMPK, p-mTOR, mTOR, p-ULK1, ULK1, LC3II, LC3I, and P62 in the abdominal adipose tissue of mice. Relative to the control group, the model cohort’s p-AMPK/AMPK ratio was markedly decreased at time points 3 (*p* < 0.01) and 9 (*p* < 0.01; Cohen’s d = 0.01; η^2^ = 1.00), with significant elevation at time points 6 and 12 (*p* < 0.01) and the lowest value at week 9. The model group’s p-mTOR/mTOR ratio was significantly elevated at time points 3 and 12 (*p* < 0.01) but markedly reduced at week 6 (*p* < 0.01) and week 9 (*p* < 0.05; Cohen’s d = 0.03; η^2^ = 0.59). The model group’s p-ULK1/ULK1 ratio was overall significantly decreased (*p* < 0.01), with no significant difference noted at week 9 (*p* > 0.05; Cohen’s d = 0.03; η^2^ = 0.42). The LC3II/LC3I ratio in the model group was significantly elevated across all time points (*p* < 0.01); at week 9, the effect size was Cohen’s d = 0.09; η^2^ = 0.99. The model group’s P62 protein expression was significantly reduced at week 3 (*p* < 0.01), pronouncedly increased at time points 9 (*p* < 0.01; Cohen’s d = 0.01; η^2^ = 0.88) and 12 (*p* < 0.01), and slightly elevated at week 6 (no statistical significance).

## 3. Discussion

The advances in cell biology and metabolic medicine have rendered the association between autophagy and hyperlipidemia a research hotspot. The role of autophagy exhibits dynamicity across different stages and tissues of hyperlipidemia. The pathogenesis of hyperlipidemia is complex, involving genetic factors, dietary patterns, lifestyle habits, and other pathogenic factors. Current therapies primarily involve inhibiting HMG-CoA reductase, activating lipoprotein lipase, stimulating autophagy-associated AMPK/mTOR signaling pathway, and reducing apolipoprotein B synthesis [20,21,22]. As a key branch of autophagy, lipophagy mainly regulates lipid metabolism by degrading intracellular lipid droplets (LDs) [23,24,25].

White adipose tissue (WAT) maintains energy homeostasis by storing or mobilizing lipids, while brown adipose tissue (BAT) produces heat through mitochondrial uncoupling. Differences in autophagic function stem from variations in lipid composition [26,27,28,29]. As the core representative of visceral white adipose tissue (vWAT) in mice, intra-abdominal white adipose tissue (IWAT) acts as a key regulatory node in the pathogenesis and progression of metabolic syndrome, and also a central mediator of hepatic lipotoxicity. Its metabolic disorder can directly induce insulin resistance and fail to buffer excess metabolic substrates under nutritional overload, thereby triggering ectopic lipid accumulation and hepatic lipotoxic injury, and ultimately forming a pathological cycle of adipose–liver metabolic dysfunction. Autophagy plays a central role in regulating lipid metabolism and adipocyte differentiation in IWAT; notably, the expression levels of autophagy-related genes and proteins in IWAT are significantly higher than those in subcutaneous white adipose tissue (sWAT), and its autophagic activity is closely correlated with plasma lipid levels and insulin resistance index. Furthermore, key molecules such as ULK1 and ATG7 can precisely regulate lipolysis, lipid oxidation, and adipogenesis in IWAT through both autophagy-dependent and autophagy-independent pathways [30,31,32]. Based on the functional specialization of the two adipose tissue types, pathological findings of adipose tissues, and the evidence from Sadatsugu Sakane et al. [33] that autophagy is upregulated in the white adipose tissue of high-fat-diet (HFD)-fed mice, combined with the unique and pivotal role of IWAT in the pathogenesis of metabolic syndrome and the mediation of hepatic lipotoxicity, IWAT (abdominal adipose tissue) was selected as the research sample in this study.

Our study showed that 9 weeks is the critical time point for metabolic disorder transition in HFD-induced ICR mice. At this point, model group mice showed synergistic changes in multiple organ indices: the liver index decreased, epididymal fat weight peaked, serum TC and LDL increased significantly, HDL began to decrease, and abdominal adipose tissue volume was reduced. These changes collectively indicate that the model has transitioned from the early stage of lipid accumulation to the metabolic compensatory phase, which is closely correlated with the dysregulation of the AMPK/mTOR axis, with mTOR inhibition at week 9 driven by a dominant AMPK-independent mechanism [34]. HFD induces sustained triglyceride and cholesterol accumulation in the liver, inducing oxidative stress and endoplasmic reticulum stress [35], and persistent endoplasmic reticulum stress acts as a key AMPK-independent trigger to directly inhibit mTOR signaling without AMPK mediation, exacerbating hepatic steatosis and pathological hyperplasia. In terms of lipid metabolism, dyslipidemia arises from the dysregulation of hepatic cholesterol synthesis and clearance, which leads to a typical lipid profile marked by increased TC and LDL levels alongside reduced HDL levels, and this disturbance is amplified by impaired mTOR-dependent lipid gene regulation under AMPK-independent conditions [36,37,38].

Adipose tissue exhibits organ-specific changes. In the early stage of HFD intervention, epididymal adipose tissue achieves compensatory lipid storage via adipocyte hypertrophy, with its organ index peaking at week 9. In contrast, abdominal adipose volume decreases, possibly due to the synergy of inflammation and autophagic impairment, among which NF-κB/TNF-α-mediated inflammatory signaling may serve as another important AMPK-independent mediator of mTOR inhibition: lipid overload induces pro-inflammatory cytokine release, activates the NF-κB/TNF-αpathway, inhibits mTOR activity, exacerbates autophagic impairment and inflammation, and ultimately leads to abdominal adipose atrophy. Notably, AMPK-independent mTOR inhibition mediated by endoplasmic reticulum stress and NF-κB/TNF-α may form a reinforcing regulatory loop in the liver and abdominal adipose tissue, aggravating lipid metabolic disorders and autophagic dysfunction. Insufficient activation of the AMPK/mTOR pathway and subsequent abnormalities may act as a core mechanism linking hepatic and adipose tissue pathologies to systemic dyslipidemia, and these pathways may be key drivers of multi-organ metabolic disturbance in mice at week 9. Accordingly, the model mice may enter a critical stage of multi-organ metabolic decompensation [39,40,41,42,43].

Inhibiting AMPK/mTOR signaling pathway regulates autophagy-related protein expression, including p-ULK1, LC3, and p62; these proteins are involved in autophagosome formation, trafficking, and lysosomal degradation, respectively. This process ultimately leads to dyslipidemia and other associated pathological changes, representing a key mechanism underlying the induction of hyperlipidemia [44,45,46,47]. Current studies have shown that AMPK regulates autophagy in a highly context-dependent manner, closely associated with the intensity and duration of metabolic stress. Under short-term mild stress, AMPK phosphorylates ULK1 to initiate protective autophagy, maintaining cellular homeostasis by degrading damaged organelles and supplementing energy; yet in sustained severe metabolic decompensation, this adaptive response shifts to an inhibitory effect [48,49,50]. Kazyken D et al. [51] found that in the sustained metabolic stress model of prolonged amino acid deprivation, AMPK failed to exert its canonical pro-autophagic effect, but instead inhibited autophagy initiation and promoted mTORC1 signaling reactivation, reversing its classic regulatory pattern on autophagy and mTOR signaling. Longo M et al. [52] further confirmed such context specificity of AMPK regulation: under energy stress, AMPK enhances PARKIN-mediated mitophagy of damaged mitochondria for cellular protection, while blocking NIX-mediated mitophagy of functional mitochondria to avoid excessive autophagic injury. In summary, extensive depletion of signaling molecule activation induced by sustained metabolic stress may abrogate AMPK’s canonical pro-autophagic function, trigger cross-regulation among multiple pathways, and ultimately exacerbate cellular metabolic disturbance.

At week 3 of the model establishment, the mice might be in the autophagy initiation phase, at which point autophagosomes start to form and autophagic flux remains normal. The p-AMPK/AMPK ratio was notably reduced, while the p-mTOR/mTOR ratio remained sharply elevated, indicating that energy surplus in early HFD feeding directly inhibits AMPK activity, thus alleviating its negative regulation of mTOR. Although mTOR overactivation theoretically suppresses ULK1, the model group’s p-ULK1/ULK1 ratio was significantly reduced; furthermore, the LC3Ⅱ/LC3Ⅰ ratio was increased and p62 protein levels were lower than the control group. This phenomenon might be attributed to an attempt at compensatory autophagy activation or transcriptional regulation that downregulates p62 expression [53,54,55].

At week 6 of the model establishment, autophagosomes continued to form in the model group, with a slight decline in their clearance efficiency. The p-AMPK/total AMPK ratio rose notably, while that of p-mTOR/total mTOR dropped in parallel, thus forming the canonical autophagy-promoting pattern characterized by AMPK activation and mTOR inhibition. This reflects adipocytes’ adaptive adjustment to lipid storage saturation: lipotoxicity induces energy stress, which then activates AMPK to counteract mTOR’s inhibition of ULK1. Although the p-ULK1/ULK1 ratio remained lower than that of the control group, sustained elevation in the LC3Ⅱ/LC3Ⅰ ratio indicated enhanced autophagy initiation; meanwhile, the expression of p62 protein exhibited a slight, non-significant upregulation (Cohen’s d = 0.01; η^2^ = 0.03), which foreshadowed the subsequent occurrence of autophagic dysfunction [56,57].

At week 9, the protein expression profiles exhibited characteristics of pivotal dysregulation, indicating a potential risk of metabolic decompensation: (1) At the signaling pathway upstream: The p-AMPK/AMPK ratio fell to its minimum, while the p-mTOR/mTOR ratio also decreased concurrently—this contradicts the canonical AMPK activation-mediated mTOR inhibition pattern. This atypical regulatory feature indicates that the intracellular signaling network has undergone significant remodeling during the stage of severe metabolic decompensation. On the one hand, extensive depletion in the activation of signaling molecules may occur, leading to the concurrent inhibition of AMPK and mTOR phosphorylation and thus abrogating their classic antagonistic regulatory relationship. On the other hand, it also demonstrates that mTOR inhibition at this stage is not AMPK-mediated but rather dominated by AMPK-independent pathways, including sustained endoplasmic reticulum stress, excessive release of inflammatory signals, metabolic or tumor-suppressive pathways directly triggered by energy insufficiency, and negative feedback regulatory mechanisms initiated by the organism under extreme metabolic disturbance. These pathways synergistically suppress mTOR activity to compensate for and alleviate cellular damage induced by metabolic decompensation. The emergence of this non-canonical regulatory pattern further underscores the severity and complexity of metabolic disturbance at this stage; the regulatory rules of a single signaling pathway can no longer explain alterations in the global signaling network, suggesting that the integrated effects of cross-regulation across multiple signaling pathways may be involved in this pathological process. (2) Autophagy initiation: The ratio of p-ULK1 to total ULK1 in the model cohort decreased sharply, implying that the control cohort initiated adaptive autophagy inhibition, whereas the model group, subjected to sustained stress, could not further downregulate ULK1 phosphorylation, with autophagy initiation functions approaching “exhaustion”. (3) Autophagic flux: A marked elevation in the LC3Ⅱ/LC3Ⅰratio was accompanied by a significant accumulation of the p62 protein, a definitive hallmark of impaired autophagic flux, indicating that dysfunction occurs at the autophagosome degradation step. Combined with the decreased p-ULK1/ULK1 ratio, these findings suggest that autophagy initiation may also be compromised. This observation is consistent with the phenomenon that lipid droplets in abdominal adipose tissue cannot be effectively cleared via the autophagic pathway, thereby resulting in abnormal lipid accumulation. Specifically, lipolysis mediated by M1-type macrophages enhances lipid mobilization, while impaired autophagic flux prevents the efficient degradation of lipid droplets, ultimately leading to their abnormal accumulation. At this stage, the extremely low AMPK activity serves as a key hub: it fails to alleviate lipotoxicity by activating autophagy, but instead exacerbates autophagic impairment, driving adipose tissue into a state of decompensation [58,59,60,61].

At week 12, the model group may exhibit a complete dysregulation of regulatory signaling, accompanied by autophagosome accumulation and impaired clearance capacity. The p-AMPK/AMPK ratio rose sharply again, while the p-mTOR/mTOR ratio increased simultaneously. This abnormal “AMPK activation unaccompanied by mTOR inhibition” pattern signals full dysregulation of the signaling pathway, likely stemming from the inflammation-driven PI3K/Akt pathway cross-activation of mTOR. At this time point, the p-ULK1/ULK1 ratio continued to decline, the LC3Ⅱ/LC3Ⅰ ratio remained elevated, and p62 expression was further significantly upregulated—thereby worsening the conflict between defective autophagy initiation and impaired clearance [62,63].

This study has the following limitations: (1) Only male ICR mice were used, which restricts the generalizability of the findings to other genders and strains; additionally, the gut microbiota, a key regulator of lipophagy and metabolic pathways, was not analyzed. (2) The concurrent decrease in the p-AMPK/AMPK and p-mTOR/mTOR ratios at week 9 was inconsistent with the classic reciprocal regulatory relationship of the AMPK–mTOR axis. Although this phenomenon was hypothesized to be associated with AMPK-independent mechanisms such as endoplasmic reticulum stress and inflammation, no direct experimental evidence was provided due to the lack of detection for endoplasmic reticulum stress markers (IRE1α, CHOP, GRP78) and inflammatory mediators (TNF-α, IL-6). (3) Conclusions regarding systemic metabolic disorders were solely derived from detections in abdominal adipose tissue without complementary analyses of the liver—the core organ for lipid metabolism. Moreover, only Oil Red O staining was used to assess hepatic lipid changes, and Western blot analysis of the AMPK/mTOR signaling pathway in liver tissue was not performed, which precluded a comprehensive reflection of the regulatory characteristics of lipid metabolism. (4) Deficiencies existed in autophagy-related detections: autophagic flux was not directly measured, and the LC3II/LC3I ratio was the only primary indicator (unable to distinguish between abnormal autophagy initiation and impaired flux). Furthermore, supplementary approaches including electron microscopy, cathepsin activity assay, and LAMP1/LAMP2 protein expression detection were not adopted to identify the specific steps and sites of abnormal autophagic flux. These limitations clarify directions for subsequent research, and the findings of this study can be further verified and extended through additional experiments.

## 4. Materials and Methods

### 4.1. Chemicals and Instruments

Hematoxylin–Eosin (HE) Staining Kit (Solarbio Science & Technology Co., Ltd., Beijing, China, Batch No. 240004005); Total Cholesterol (T-TC) Assay Kit, Triglycerides (TG) Assay Kit (Nanjing Jiancheng Institute of Bioengineering, Nanjing, China, Batch Nos. A110-1-1 and A111-1-1, respectively); Biochemical Detection Kits for Triglycerides (TG), Total Cholesterol (TC), LDL Cholesterol (LDL-C), and HDL Cholesterol (HDL-C) (BioSino Bio-Technology and Services Co., Ltd., Beijing, China, Batch Nos. 232091, 248131, 231841, and 231871, respectively); 2nd-Generation Reverse Transcription Kit, Total RNA Extraction Kit for Animal Tissues/Cells, SYBR Green qPCR Master Mix (Wuhan Servicebio Biotech Co., Ltd.; Wuhan, China, SBT, Batch Nos. MPC2405029, MPC2405043, and MPC2405046, respectively); anti-fluorescence quenching mounting medium (containing DAPI) (Servicebio Technology Co., Ltd., Wuhan, China, Batch No. G1407-25ML); FITC-conjugated goat anti-rabbit IgG (Cat. No. GB22303); primary antibodies against AMPK, phosphorylated AMPK (p-AMPK), ULK1 and p-ULK1 (Wanleibio Technology Co., Ltd., Shenyang, China, Batch Nos. WL02254, WL05103, WL03067 and WLA0369, respectively); primary antibodies against mTOR, phosphorylated mTOR (p-mTOR), LC3 and P62; recombinant HRP-labeled anti-mouse IgG (H + L) goat antibody; recombinant HRP-labeled anti-rabbit IgG (H + L) goat antibody (Proteintech, Wuhan, China), Batch Nos. 66888-1-Ig, 67778-1-Ig, 14600-1-AP, 84826-1-RR, RGAM001, and RGAR001, respectively); primary antibodies against β-actin and Beclin-1 (Biosynthesis Biotechnology Co., Ltd., Beijing, China, Batch Nos. bs-0061R and bsm-41365R, respectively).

Inverted microscope (Olympus Corporation, Tokyo, Japan; Model: IX73); microplate reader (Tecan Group Ltd., Männedorf, Switzerland; Model: Infinite 200 PRO); automatic biochemical analyzer (Hitachi, Ltd., Tokyo, Japan; Model: 7600-020); gel imaging system (GE Healthcare, Chicago, IL, USA; Model: Amersham Imager 600). JH-32 square-faced hand press (Sheng Yue Mechanical Equipment Co., Ltd., Jinhua, China).

### 4.2. Laboratory Animals

Eighty male ICR mice were procured from Liaoning Changsheng Biotechnology Co., Ltd., Shenyang, China, (animal use license: SCXK (Liao) 2020-0001). All rodents were maintained in a regulated environment (20–24 °C; 50–60% relative humidity) with ad libitum access to a standard solid diet and drinking water. All animal experimental protocols were reviewed and formally endorsed by the Laboratory Animal Ethical Review Board of Heilongjiang University of Chinese Medicine (Approval No. 2025030521), and the procedures were performed in strict adherence to the ethical standards for animal research throughout the entire experiment.

### 4.3. Feed Preparation

Normal Chow Diet: Mice were fed a normal chow diet procured from Liaoning Changsheng Biotechnology Co., Ltd. (Production License: SCXK (Liao) 2020-0002; Batch No.: 23101611). The product’s constituents encompass maize, soyabean meal, wheat starch, wheat middlings, fish protein concentrate, table salt, calcium orthophosphate, limestone flour, multivitamins, multiminerals, amino acids, and other components. The diet was processed into pellets using a JH-32 square-headed hand press in combination with a tablet press under constant pressure, followed by a drying procedure. In accordance with the specification requirements, the maintenance diet was placed in a cylindrical grinding device with an inner diameter of 3 cm for pulverization, and each prepared pellet weighed around 19 g.

Self-Made High-Fat Diet: The recipe was composed of 67% murine husbandry diet, 10% pork fat, 20% table sugar, 2.5% dietary cholesterol, and 0.5% cholic acid sodium salt. The mouse maintenance diet was first ground using a grinder, after which lard, sucrose, cholesterol, and sodium cholate were added in sequence in line with the abovementioned ratios. After thorough mixing, the blended mixture was loaded into a cylindrical grinding device featuring an internal diameter of 3 cm. The mixture was then tableted and dried under constant pressure using a JH-32 square-faced manually operated press, with each prepared feed pellet weighing approximately 19 g.

### 4.4. Establishment of Hyperlipidaemic Murine Model

Mice were bred in five separate batches for this study, with 16 male ICR experimental mice selected every three weeks, resulting in a total of 80 mice enrolled. Based on the effect size of lipid metabolism indicators in preliminary experiments, the sample size of 8 mice per group was determined via power analysis (with a significance level of α = 0.05 and a statistical power of 1 − β = 0.8), which ensured sufficient statistical power to detect potential intergroup differences. The sample size selection in this study also referred to relevant experimental studies on hyperlipidemia models [64,65]. Mice in each batch were randomly and equally divided into the control group (CG) and the model group (MG), with 8 mice per group; randomization was performed using a random number table to ensure the balance of baseline characteristics (e.g., body weight) among all groups. According to the differences in feeding durations, the groups were labeled as the 15-week control group (15W-CG), 15-week model group (15W-MG), 12-week control group (12W-CG), 12-week model group (12W-MG), 9-week control group (9W-CG), 9-week model group (9W-MG), 6-week control group (6W-CG), 6-week model group (6W-MG), 3-week control group (3W-CG), and 3-week model group (3W-MG), respectively. Every mouse in each group was given free availability of drinking water over the full course of the experiment. Mice allocated to the CG were provided with the basal maintenance diet throughout the experimental period, whereas those in the MG were continuously supplied with the homemade high-fat diet (HFD). Experimental samples of all groups were uniformly harvested at the conclusion of the 15th week of feeding for subsequent index assays.

### 4.5. Observation of General Conditions of Mice

The hair status, locomotor activity, and diarrheal manifestations of the mice were monitored daily.

### 4.6. Mouse Body Weight

Murine body weight within each group was regularly documented on a weekly basis.

### 4.7. Determination of Organ Indices

Intact hepatic tissue, scapular adipose pad, epididymal adipose depot, and abdominal adipose pad were dissected and collected. These tissues were rinsed repeatedly with physiological saline, blotted dry using filtering paper to blot away surface moisture, and weighed, and their weight data were recorded. The organ index of each sample was then calculated per the following:

Formula: Organ Index (%) = [Body Weight (g)/Organ Weight (g)] × 100%

### 4.8. Quantification of Serum Biochemical Indices in Mice

Murine were anesthetized by virtue of intraperitoneal (i.p.) administration of pentobarbital sodium. Blood specimens were harvested promptly from the retro-orbital venous plexus into Eppendorf (EP) tubes, followed by centrifugation at 1500× *g* for 10 min to separate serum. Serum concentrations of TC, TG, HDL-C, and LDL-C were measured with an automated biochemic analyzer.

### 4.9. Quantification of TC and TG Levels Within Hepatic Tissue

Exactly 0.1 g of hepatic parenchyma was weighed out and combined with 9 volumes of homogenization buffer. Thorough homogenization of the mixture was conducted with a mechanical tissue homogenizer in an ice-water bath to prepare liver homogenate. Levels of TC and TG in the homogenate were measured in strict accordance with the instructions provided by the assay kits.

### 4.10. Oil Red O Lipid Stain and Hematoxylin–Eosin (H&E) Morphological Staining

Lipid-Specific Oil Red O Staining: Hepatic tissues were encapsulated using an OCT compound, then sectioned into 8-micron-thick slices, treated with Oil Red O lipophilic staining reagent, rinsed thoroughly, and finally mounted using neutral resin.

H&E: Following mouse euthanasia, abdominal, epididymal, and scapular adipose tissue samples were harvested, immobilized with 4% paraformaldehyde (PFA) solution for over 24 h, and infiltrated into paraffin resin. Subsequently, the paraffin blocks were microtomed and dyed using H&E staining reagent, and digital scanning of the tissue section specimens was performed for morphological analysis.

### 4.11. Immunofluorescence Staining of P-AMPK, P-ULK1, and Beclin-1 in Mouse Abdominal Adipose Tissue

Visceral abdominal adipose tissue specimens were harvested from mice, fixed in 4% paraformaldehyde (PFA) solution, and processed for routine paraffin embedding. One mouse was assigned to each group, and the abdominal adipose tissue of each individual mouse was embedded independently into a single paraffin block. The paraffin blocks were then serially sectioned into 2 μm thick histological slices, and 3 fields of view were observed for each tissue slice. The sections were blocked using 5% BSA blocking buffer for 10 min and subsequently incubated overnight at 4 °C in a humidified chamber with primary antibodies targeting p-AMPK, p-ULK1, and Beclin-1 (1:150, 1:100, and 1:50 dilutions, respectively). After rinsing, the tissue section specimens were incubated with biotinylated secondary antibody (1:200 dilution) at room temperature in the dark for 1 h. Finally, the sections were counterstained, mounted with anti-fluorescence quenching mounting medium, observed under an inverted microscope (Olympus Corporation, Tokyo, Japan; Model: IX73) at 400× magnification, and subjected to quantitative analysis using ImageJ 1.8.0.345 software with a preset fluorescence intensity threshold of 5.

### 4.12. Western Blotting Analysis of AMPK/mTOR Signaling Pathway-Related Proteins in Mouse Abdominal Adipose Tissue

Abdominal adipose tissues were collected from rats in each group. Core steps comprised RIPA lysis and BCA protein quantification for concentration assessment, followed by target protein visualization denaturation, electrophoresis, membrane transfer, and blocking. Nitrocellulose membranes were incubated overnight at 4 °C with specific primary antibodies targeting p-AMPK (1:500), AMPK (1:1000), p-mTOR (1:2000), mTOR (1:5000), p-ULK1 (1:500), ULK1 (1:1000), LC3 (1:1000), P62 (1:5000), and β-Actin (1:1000). Subsequently, the membranes were rinsed through three rounds of washing with phosphate buffer saline (PBS) and then incubated with secondary antibody (1:1000) at RT for 2 h, after which an additional five PBS rinses were performed. Enhanced chemiluminescence (ECL) reagent was evenly dispensed onto the membranes, and signals were imaged. β-actin served as the internal control, and grayscale analysis was conducted via ImageJ software.

### 4.13. Quantitative Data Statistical Evaluation

Experimental data were subjected to statistical analysis using GraphPad Prism 10.6.1 and expressed as mean ± standard deviation (SD). Intergroup differences were assessed via unpaired Student’s *t*-test, and statistical significance was established using SPSS Statistics v.31.0 (IBM Corporation, Armonk, NY, USA).

## 5. Conclusions

**Figure 12:**
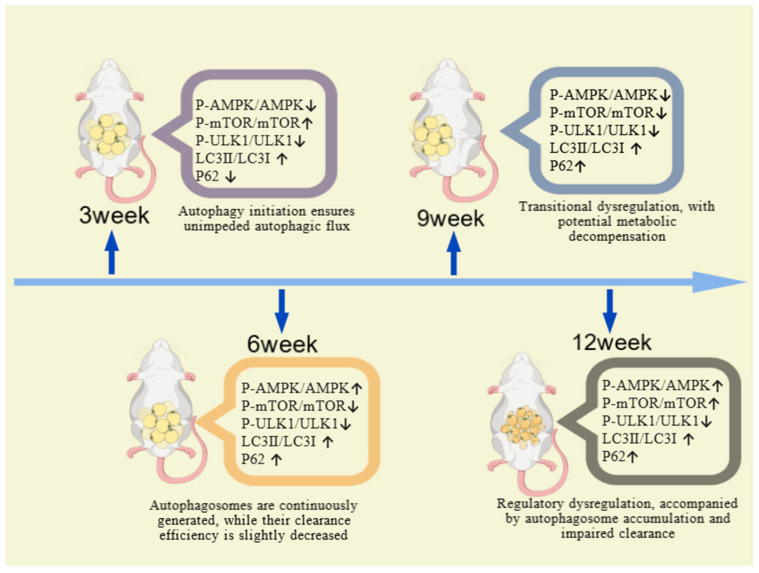
Dynamic changes in autophagy at different modeling durations created with BioGDP.com [12].

In summary, the dynamic changes in autophagy-related proteins in abdominal adipose tissue are as shown in Figure 12. The time-dependent dysregulation of the AMPK/mTOR pathway is the core mechanism underlying adipose metabolic disorders. During 3–6 weeks of HFD feeding, the mice may be in a regulable stage of mild metabolic disorder; during 12–15 weeks, they may progress to a severe stage of complete metabolic disorder. Notably, week 9 represents the critical time point when the pathway shifts from “compensatory adjustment” to “decompensatory dysregulation”—impaired, autophagic flux caused by decreased AMPK activity is the direct molecular basis for the “enhanced lipolysis yet abnormal lipid accumulation” in abdominal adipose tissue. This further validates the scientific rationale of week 9 as the “metabolic watershed” in the HFD-induced hyperlipidemic ICR mouse model, providing a robust reference for subsequent intervention studies targeting the AMPK pathway.

## Data Availability

The original contributions presented in this study are included in the article/Supplementary Material. Further inquiries can be directed to the corresponding authors.

## Supplementary Materials

The following supporting information can be downloaded at: https://www.mdpi.com/article/10.3390/ijms27031573/s1.
