# Supplementary material for: Lipophagy Dynamics in Hyperlipidemia Model ICR Mice Across Different High-Fat-Diet Feeding Durations
Source: Int J Mol Sci. 2026 Feb 5;27(3):1573. doi: 10.3390/ijms27031573 (PMC12898429; doi:10.3390/ijms27031573)
Supplement: Supplementary file 1 [file ijms-27-01573-s001.zip › Table S1 Effect size among groups.pdf]

Table S1 Effect size among groups

Body Weight in Mice		
group	Cohen's d	η^2
3Con vs 3Mod	1.69773	0.35
6Con vs 6Mod	3.47258	0.34
9Con vs 9Mod	6.08350	0.43
12Con vs 12Mod	3.99238	0.53
15Con vs 15Mod	5.41147	0.30
Hepatic organ coefficient		
group	Cohen's d	η^2
3Con vs 3Mod	0.613655	0.44
6Con vs 6Mod	0.597998	0.21
9Con vs 9Mod	0.608529	0.05
12Con vs 12Mod	0.390317	0.32
15Con vs 15Mod	0.447019	0.23
Epididymal adipose tissue index		
group	Cohen's d	η^2
3Con vs 3Mod	0.375637	0.69
6Con vs 6Mod	0.365885	0.89
9Con vs 9Mod	0.670418	0.88
12Con vs 12Mod	0.526754	0.89
15Con vs 15Mod	0.592724	0.86
Abdominal adipose coefficient		
group	Cohen's d	η^2
3Con vs 3Mod	0.322855	0.44
6Con vs 6Mod	0.415515	0.39
9Con vs 9Mod	0.577846	0.42
12Con vs 12Mod	0.435574	0.60
15Con vs 15Mod	0.493437	0.59
Scapular adipose tissue coefficient		
group	Cohen's d	η^2
3Con vs 3Mod	0.166738	0.61
6Con vs 6Mod	0.177244	0.54
9Con vs 9Mod	0.145700	0.77
12Con vs 12Mod	0.302324	0.56
15Con vs 15Mod	0.289181	0.58
TC(mmol/L)		
group	Cohen's d	η^2
3Con vs 3Mod	0.620099	0.51
6Con vs 6Mod	0.588359	0.54
9Con vs 9Mod	0.435262	0.91
12Con vs 12Mod	0.540259	0.15
15Con vs 15Mod	0.602822	0.82
TG(mmol/L)		

group	Cohen's d	η^2
3Con vs 3Mod	0.336598	0.64
6Con vs 6Mod	0.301406	0.04
9Con vs 9Mod	0.688308	0.39
12Con vs 12Mod	0.297022	0.92
15Con vs 15Mod	0.531857	0.53

HDL(mmol/L)

group	Cohen's d	η^2
3Con vs 3Mod	0.217587	0.93
6Con vs 6Mod	0.246679	0.65
9Con vs 9Mod	0.294977	0.56
12Con vs 12Mod	0.348708	0.15
15Con vs 15Mod	0.471349	0.61

LDL(mmol/L)

group	Cohen's d	η^2
3Con vs 3Mod	0.046211	0.95
6Con vs 6Mod	0.033906	0.97
9Con vs 9Mod	0.066764	0.86
12Con vs 12Mod	0.034163	0.85
15Con vs 15Mod	0.064843	0.67

TC(mmol/g)

group	Cohen's d	η^2
3Con vs 3Mod	0.513314	0.83
6Con vs 6Mod	0.669024	0.54
9Con vs 9Mod	0.576403	0.63
12Con vs 12Mod	0.387961	0.64
15Con vs 15Mod	0.332013	0.85

TG(mmol/g)

group	Cohen's d	η^2
3Con vs 3Mod	0.971799	0.08
6Con vs 6Mod	0.753234	0.12
9Con vs 9Mod	0.552950	0.18
12Con vs 12Mod	0.734861	0.28
15Con vs 15Mod	1.216306	0.03

P-AMPKT^{172}(IF)

group	Cohen's d	η^2
9Con vs 9Mod	3.035187	0.98
12Con vs 12Mod	3.098065	0.98
15Con vs 15Mod	3.732529	0.37

P-ULK1^{757}(IF)

group	Cohen's d	η^2
9Con vs 9Mod	1.883997	0.97
12Con vs 12Mod	2.611417	0.94

15Con vs 15Mod	1.558480	0.64
	Beclin-1(IF)	
group	Cohen's d	η^2
9Con vs 9Mod	5.169026	1.00
12Con vs 12Mod	5.647010	0.96
15Con vs 15Mod	0.528152	1.00
	P-AMPK^{T172}/AMPK(WB)	
group	Cohen's d	η^2
3Con vs 3Mod	0.051366	0.92
6Con vs 6Mod	0.038054	0.89
9Con vs 9Mod	0.011370	1.00
12Con vs 12Mod	0.032759	0.82
	P-mTOR^{2448}/mTOR(WB)	
group	Cohen's d	η^2
3Con vs 3Mod	0.014528	0.96
6Con vs 6Mod	0.036616	0.81
9Con vs 9Mod	0.032052	0.59
12Con vs 12Mod	0.030898	0.99
	P-ULK1^{757}/ULK(WB)	
group	Cohen's d	η^2
3Con vs 3Mod	0.067707	0.91
6Con vs 6Mod	0.036161	0.99
9Con vs 9Mod	0.030293	0.42
12Con vs 12Mod	0.046760	0.92
	LC3II/I(WB)	
group	Cohen's d	η^2
3Con vs 3Mod	0.126084	0.94
6Con vs 6Mod	0.033269	0.99
9Con vs 9Mod	0.087128	0.99
12Con vs 12Mod	0.118855	0.99
	P62(WB)	
group	Cohen's d	η^2
3Con vs 3Mod	0.011178	0.89
6Con vs 6Mod	0.007437	0.03
9Con vs 9Mod	0.009758	0.88
12Con vs 12Mod	0.024695	0.99
