# Supplementary material for: Lipophagy Dynamics in Hyperlipidemia Model ICR Mice Across Different High-Fat-Diet Feeding Durations
Source: Int J Mol Sci. 2026 Feb 5;27(3):1573. doi: 10.3390/ijms27031573 (PMC12898429; doi:10.3390/ijms27031573)
Supplement: Supplementary file 1 [file ijms-27-01573-s001.zip › Table S2 Initial body weight of experimental mice in each group.pdf]

Table S2 Initial body weight of experimental mice in each group (Mean±SD, n=8)

| Group | Initial body weight |
|---|---|
| 3Con | 19.93±0.74 |
| 3Mod | 20.81±1.32 |
| 6Con | 19.82±1.74 |
| 6Mod | 19.57±1.70 |
| 9Con | 18.87±1.22 |
| 9Mod | 18.81±1.08 |
| 12Con | 19.38±1.47 |
| 12Mod | 20.02±1.41 |
| 15Con | 18.92±1.29 |
| 15Mod | 18.75±1.25 |
