# Supplementary material for: Lipophagy Dynamics in Hyperlipidemia Model ICR Mice Across Different High-Fat-Diet Feeding Durations
Source: Int J Mol Sci. 2026 Feb 5;27(3):1573. doi: 10.3390/ijms27031573 (PMC12898429; doi:10.3390/ijms27031573)
Supplement: Supplementary file 1 [file ijms-27-01573-s001.zip › Table S3 Initial Blood Lipid Levels.pdf]

Table S3 Initial Blood Lipid Levels

group	TC(mmol/L)	TG(mmol/L)	HDL(mmol/L)	LDL(mmol/L)
3Con	2.18±0.26	1.40±0.20	1.72±0.14	0.34±0.09
3Mod	2.24±0.17	1.52±0.25	1.64±0.20	0.28±0.06
6Con	2.40±0.33	1.57±0.28	1.71±0.07	0.35±0.07
6Mod	2.27±0.37	1.48±0.24	1.58±0.18	0.33±0.25
9Con	2.31±0.35	1.46±0.23	1.70±0.08	0.37±0.06
9Mod	2.34±0.62	1.52±0.24	1.66±0.08	0.31±0.07
12Con	2.22±0.20	1.48±0.27	1.65±0.14	0.32±0.12
12Mod	2.14±0.21	1.56±0.23	1.69±0.09	0.35±0.09
15Con	2.25±0.27	1.49±0.21	1.71±0.09	0.29±0.06
15Mod	2.20±0.24	1.50±0.19	1.70±0.08	0.33±0.10
