# Supplementary figures and images for: Lipophagy Dynamics in Hyperlipidemia Model ICR Mice Across Different High-Fat-Diet Feeding Durations
Source: Int J Mol Sci. 2026 Feb 5;27(3):1573. doi: 10.3390/ijms27031573 (PMC12898429; doi:10.3390/ijms27031573)

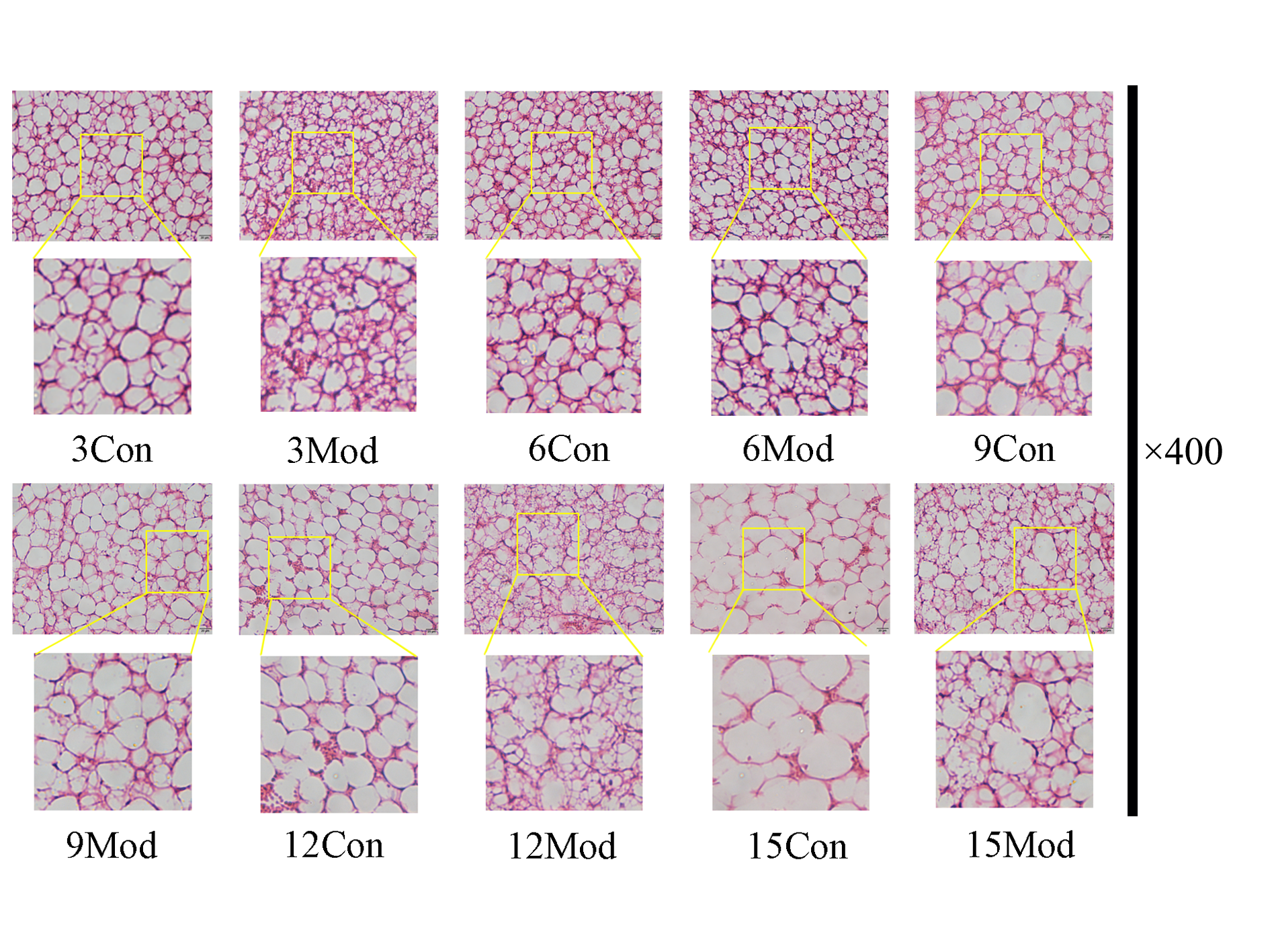

Supplement: Supplementary file 1 [file ijms-27-01573-s001.zip › Magnified local views of histological images/BWAT HE.png]

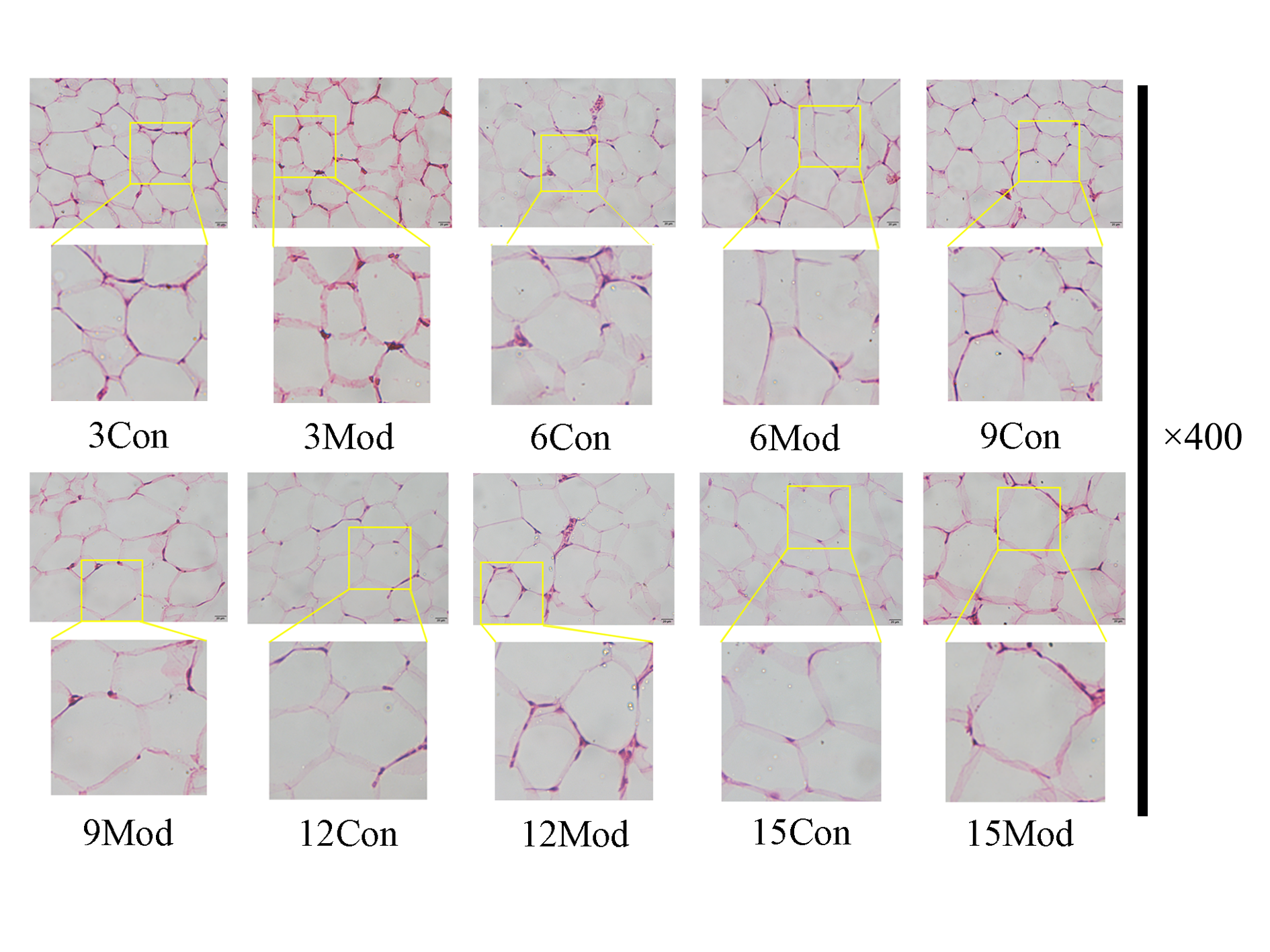

Supplement: Supplementary file 1 [file ijms-27-01573-s001.zip › Magnified local views of histological images/EWAT HE.png]

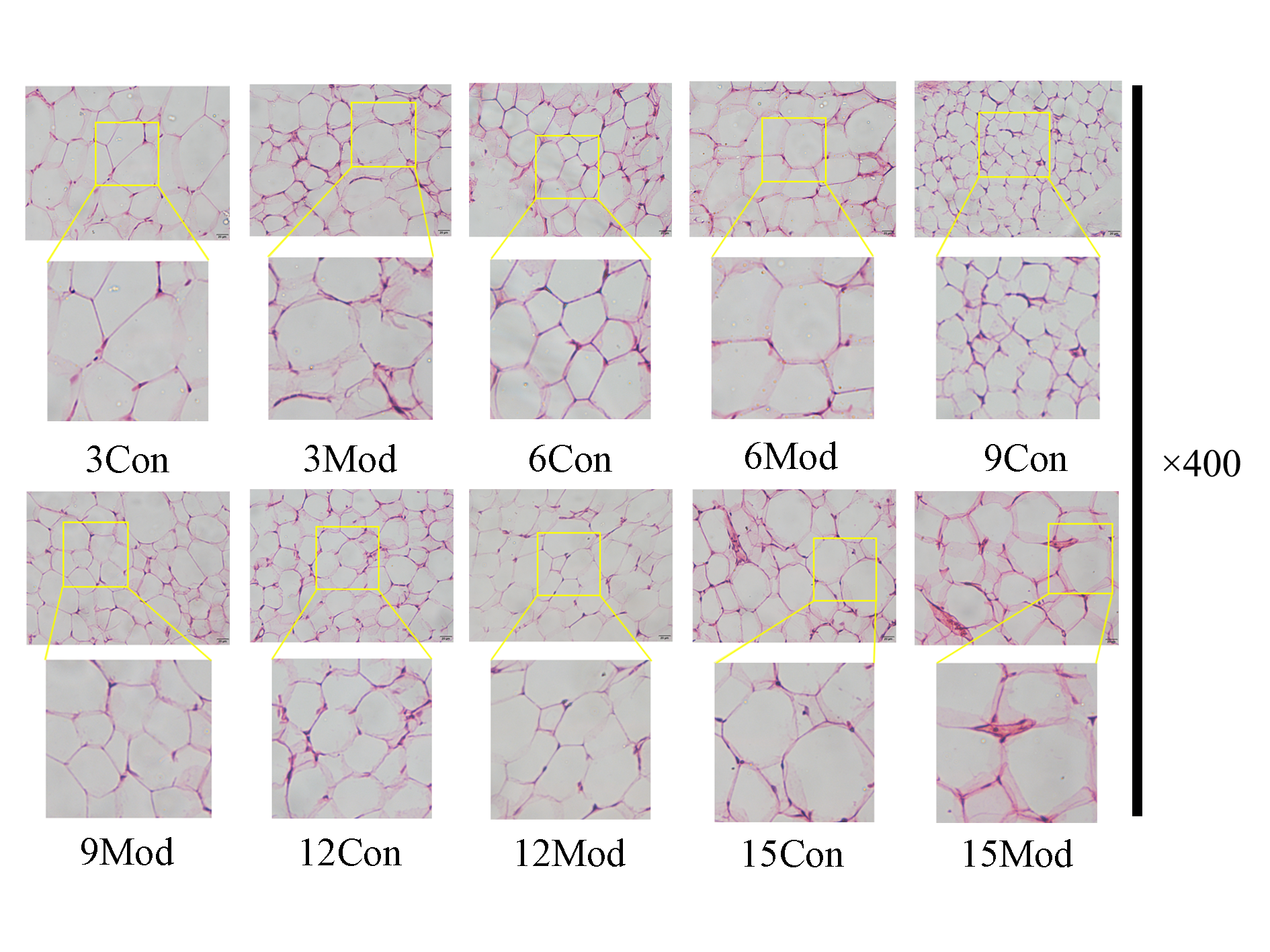

Supplement: Supplementary file 1 [file ijms-27-01573-s001.zip › Magnified local views of histological images/IWAT HE.png]

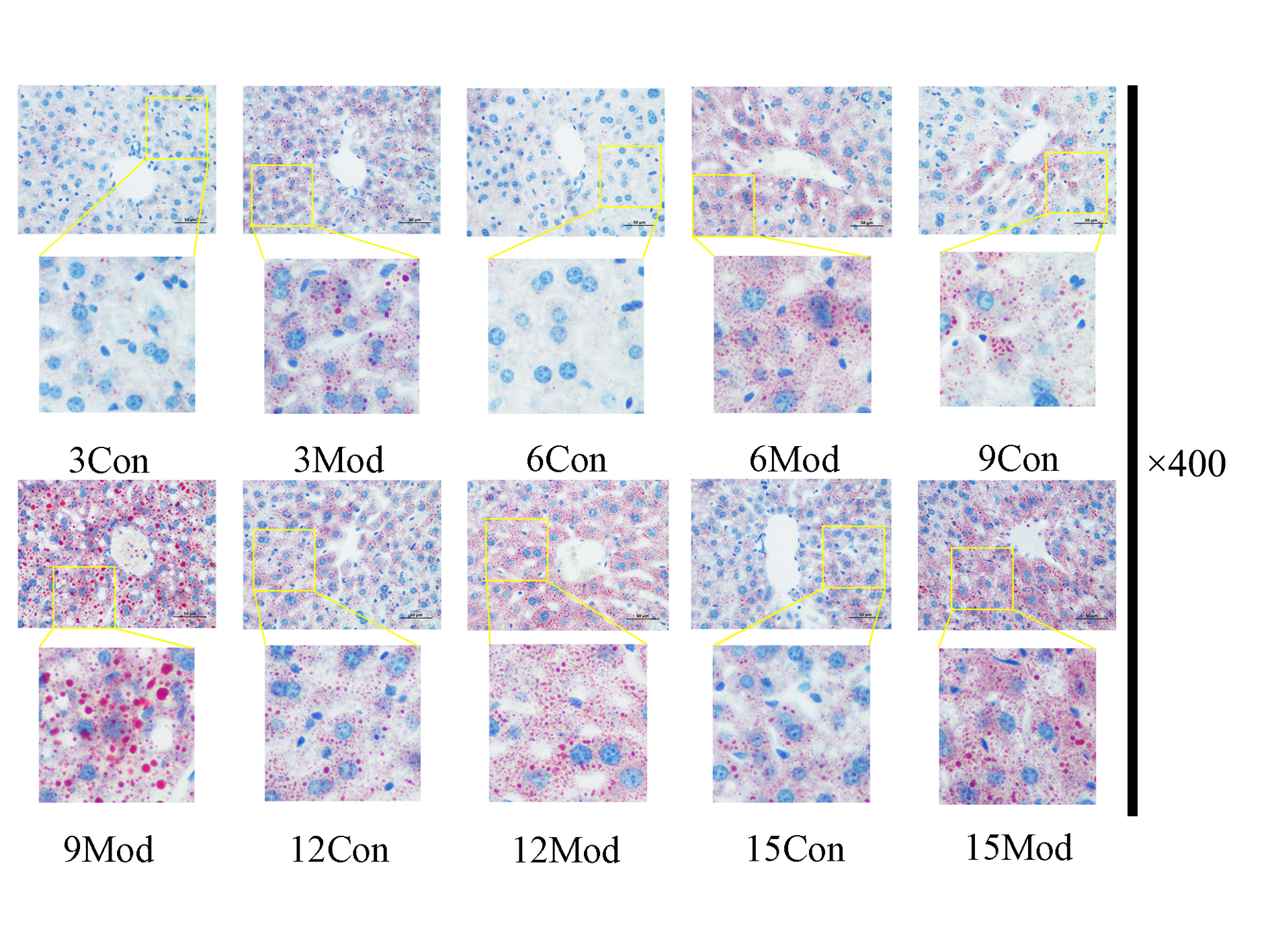

Supplement: Supplementary file 1 [file ijms-27-01573-s001.zip › Magnified local views of histological images/Oil Red O staining of mouse liver.png]
